# Qualitative aspects of learning, recall, and recognition in dementia

**DOI:** 10.4103/0972-2327.64639

**Published:** 2010

**Authors:** Neelima Ranjith, P. S. Mathuranath, Gangadhar Sharma, Aley Alexander

**Affiliations:** Cognition and Behavioral Neurology Section (CBNS), Department of Neurology, Sree Chitra Tirunal Institute for Medical Sciences and Technology (SCTIMST), Trivandrum - 695020, Kerala, India

**Keywords:** Serial position effect, Alzheimer's disease, vascular dementia, frontotemporal dementia

## Abstract

**Objective:**

To determine whether learning and serial position effect (SPE) differs qualitatively and quantitatively among different types of dementia and between dementia patients and controls; we also wished to find out whether interference affects it.

**Materials and Methods:**

We administered the Malayalam version of the Rey Auditory Verbal Learning Test (RAVLT) to 30 cognitively unimpaired controls and 80 dementia patients [30 with Alzheimer's disease (AD), 30 with vascular dementia (VaD), and 20 with frontotemporal dementia (FTD)] with mild severity on the Clinical Dementia Rating Scale.

**Results:**

All groups were comparable on education and age, except the FTD group, who were younger. Qualitatively, the learning pattern and SPE (with primacy and recency being superior to intermediate) was retained in the AD, VaD, and control groups. On SPE in free recall, recency was superior to intermediate in the FTD group (*P* < 0.01 using Bonferroni correction). On recognition, the AD and VaD groups had more misses (*P* < 0.01), while the FTD group had more false positives (*P* < 0.01).

**Conclusion:**

Quantitative learning is affected by dementia. The pattern of qualitative learning remains unaltered in dementia in the early stages.

## Introduction

Learning is a process by which information is acquired; it forms the background against which information is encoded and stored in memory. The process of learning and recall involves a definite pattern. The study of this pattern in the healthy and the diseased can enhance our understanding of the organization of memory in the brain and the effect of disease on it.

The Rey Auditory Verbal Learning Test (RAVLT)[1,2] is a widely used and well-validated word-list memory test that has been used extensively in assessing the learning curve, strength of memory after interference task, and pattern of learning (serial positioning effects), as well as for measuring recognition memory. Normally, during free recall of a series of unrelated words from a word list, individuals tend to follow a pattern known as the serial position effect (SPE), i.e., words from the beginning (primacy effect) and the end (recency effect) of the list are recalled better than the mid-list (intermediate) items.[3] There is considerable data available on the patterns of learning in healthy individuals, and the pattern of SPE has been shown to be relatively preserved in the healthy elderly.[4] However, little is known about what happens to the learning pattern in patients with dementia. Pepin and Eslinger[5] in their study reported that patients with mild Alzheimer's disease (AD) demonstrated a 'U' shaped serial position curve similar to that observed in non-demented individuals. Buschke *et al*.[6] studied 18 patients with mild AD and 231 individuals with no cognitive impairment and demonstrated that mild AD patients had impaired primacy compared to cognitively unimpaired individuals. Based on their study on 32 patients with AD, 20 patients with depression, and 18 normal individuals, Foldi *et al*.[7] showed that AD patients demonstrated a recency effect. Paul *et al*.,[8] in their study on vascular dementia (VaD) patients, reported that individuals with mild VaD demonstrated intact SPE.

There are a few published studies on the qualitative aspects of memory in patients with mild dementia. Most of the published studies are on memory, rather than on learning and the pattern of recall.

In the present study we investigate the qualitative aspects of learning and recall in elderly patients with dementia. Using RAVLT translated into the local language, Malayalam, we studied the learning pattern of patients with different types of dementia – AD, VaD, and frontotemporal dementia (FTD) – and compared it with that of cognitively unimpaired controls. Our objectives were: a) to determine if the learning pattern in patients with dementia differs from that in controls and b) to compare and contrast the effects of interference on SPE in patients with dementia and in controls. We hypothesized that the dementia group would differ in their performance from the controls not only quantitatively, i.e., in the number of words recalled, but also qualitatively, i.e., in the pattern of learning and recall.

## Materials and Methods

### Study design and participants

The design that we have employed in our study is a correlational one. The study patients were from the memory clinic of the hospital and included patients with AD (*n* = 30), VaD (*n* =30), and FTD (*n* =20), who had been diagnosed using the standard international criteria of National Institute of Neurological and Communicative Disorders and Stroke-Alzheimer's Disease and Related Disorders Association (NINCDS–ADRDA),[9] National Institute of Neurological Disorders and Stroke-Association Internationale pour la Recherche et l'Enseignement en Neurosciences (NINDS–AIREN),[10] and the consensus criteria,[11] respectively; for comparison we also had cognitively unimpaired healthy controls (*n* =30). The diagnosis and the dementia subtyping of patients attending the memory clinic was finalized in a consensus conference, involving the neurologists, psychiatrists, and neuropsychologists, and using the clinical, biochemical, neuroimaging, and neuropsychological data. The consensus conference was blinded to the RAVLT data of the patients participating in the study. The cognitively unimpaired participants were selected from the community after a clinical evaluation and screening using the Addenbrooke's Cognitive Examination,[12,13] Instrumental Activities of Daily Living in Elderly,[14] Hospital Anxiety and Depression Scale,[15] and Clinical Dementia Rating Scale.[16] The AD and VaD groups were comparable with the controls on age. The FTD group, however, as expected, was younger. All the four groups were comparable on education [Table 1]. All patients had disease of mild severity on the Clinical Dementia Rating Scale (CDR~1). All participants were native speakers of Malayalam, a Dravidian language of South India. All participants gave informed voluntary consent to participate in the study, which had received the approval of the Institute Ethics Committee and was carried out in compliance with the regulations of our institution.

**Table 1:** Demographic details and scores on RAVLT

	Control (n=30)	AD (n = 30)	VaD (n = 30)	FTD (n=20)	*P* value
	Mean ± SD	Mean ± SD	Mean ± SD	Mean ± SD
Age	65.47 ± 7.86	68.93 ±7.28	64.67 ± 10.54	60.65 ± 11.13	0.022
Education	10.87 ±13.92	10.17 ± 4.61	12.93 ± 11.27	11.75 ± 5.16	0.731
CDR Total		1.05 ± 0.53	0.98 ± 0.46	0.93 ± 0.52	0.861
Trial 1	3.57 ±1.91	2.30 ± 1.49	2.77 ± 1.57	2.90 ± 1.89	0.043
Trial 2	5.90 ±2.23	3.17 ± 1.80	4.00 ± 1.86	4.10 ± 1.52	0.000
Trial 3	6.83 ±2.00	3.70 ± 2.17	4.20 ± 2.11	4.45 ± 1.91	0.000
Trial 4	7.77 ±2.46	4.17 ± 2.23	4.17 ± 2.18	4.80 ± 1.96	0.000
Trial 5	8.17 ±2.36	4.30 ± 2.44	4.80 ± 3.16	5.25 ± 2.15	0.000
Immediate recall (pre-interference)	6.45 ± 1.84	3.53 ± 1.81	3.99 ± 1.98	4.30 ± 1.46	0.000
Immediate recall (post-interference)	5.17 ± 2.89	1.13 ± 1.38	1.93 ± 2.38	2.55 ± 2.76	0.000
Delayed	5.80 ± 2.90	0.23 ± 0.77	0.83 ± 1.74	2.15 ± 3.15	0.000
Recognition	12.40 ± 2.43	6.97 ± 5.05	7.17 ± 5.12	11.30 ± 5.14	0.000
Hit rate	0.827 ± 0.162	0.464 ± 0.337	0.478 ± 0.395	0.753 ± 0.342	0.000
Misses	0.173 ± 0.162	0.536 ± 0.337**	0.529 ± 0.389**	0.260 ± 0.337	0.000
False positives	0.134 ± 0.164	0.268 ± 0.303	0.244 ± 0.341	0.528 ± 0.424*	0.000
Recognition d-Prime	2.37 ± 0.662	0.731 ± 0.531	0.878 ± 0.732	0.721 ± 1.036	0.000

Test used- Multivariate ANOVA. In the recognition part of RAVLT

* FTD had significantly more false positives than controls and

** AD and VaD had significantly more misses than controls

### Tools and administration

The Malayalam version of the RAVLT was administered to all participants by a trained neuropsychologist, a native speaker of Malayalam. The items in the original RAVLT were translated into Malayalam by a linguist proficient in both English and Malayalam. To validate the interpretation it was back-translated by another person who was also proficient in both languages. Due to the lack of a Malayalam equivalent for the word 'ranger,' the word 'carpenter' was used in place of the word 'ranger.' The word 'carpenter' has a Malayalam equivalent and has high imageability. Because no published word-frequency data was available for the Malayalam words, we chose words based on colloquial frequency. For the recognition task, along with the words in list A and B, phonemic and semantic distracters were prepared.

The participants were read out the list of words (list A) at a pace of one word per second. The scores obtained on trials 1 to 5 were used as a measure of learning and the scores obtained on the fifth trial as a measure of the pre-interference immediate recall. The distracter list (list B) was then read out and the participant was asked to recall it. Following this, the participant was asked to recall the words from list A; this was used as a measure of post-interference free recall. The delayed recall task was administered after 20 min and was followed by the recognition task in which the examiner read aloud a list of 50 words (this list included words from both list A and B and words phonemically or semantically related to them) from which the participants had been instructed to identify the words in list A.

### Scoring

The total number of correct responses in each trial and the order of word recall in the first trial, immediate pre-interference, immediate post-interference, and delayed recall trials were recorded on the scoring sheet. On the recognition task, 'hits' refer to the number of words correctly identified from list A, and 'misses' refer to the words from list A that were not identified. The words incorrectly identified as present in list A constitute the false positives.

To study the SPE, we looked into the pattern of recall for all the 15 items in the list. Further, we divided the entire list of 15 words into five parts of three words each—the first three words representing primacy, the middle three words representing intermediate, and the last three words representing recency. We used the scores on trial 1 to study SPE in free recall.

### Statistics

ANOVA was used to compare the means across the different groups. *Post hoc* analysis was done using the Bonferroni test. The paired t-test was used for intra-group comparison of performance. Wherever multiple comparisons were done, a Bonferroni correction was applied for determining the significance of the *P* value. A recognition discriminability index was calculated using the d-prime analysis.

## Results

There was significant difference (*P* < 0.01) between the normal controls and the dementia groups in the mean number of words recalled in each of the five free recall trials, the immediate recall of words in the pre interference and post-interference trials, in delayed recall and in recognition trials [Table 1]. Although the rate of learning was lower for dementia patients, they followed the same pattern of learning as that of normal controls across the five trials, i.e., improvement in the number of words recalled with each subsequent trial. When the three dementia groups were compared with one another, there was no significant difference (*P* > 0.05) between the groups in any of the parameters of memory.

All the dementia groups performed better on the recognition task than on the delayed-recall task (*P* = 0.05). The d-prime value showed that controls demonstrated better recognition of words from list A than the dementia patients. The scores of the patient groups indicated that within the dementia groups, the VaD group performed better on recognition than the AD group, while the AD group performed better than the FTD group, although the differences were not statistically significant.

With regard to the type of errors on recognition, the AD and VaD groups demonstrated more misses than the control and the FTD groups (*P* < 0.01). The FTD group demonstrated significantly more false positives than the controls or the other two dementia groups (*P* < 0.01). Within-group comparison showed that the AD and VaD groups demonstrated significantly more misses than false positives (*P* < 0.05). However, in the FTD group, the hits and false positives did not show a statistically significant difference (*P* > 0.05).

**Figure 1:**
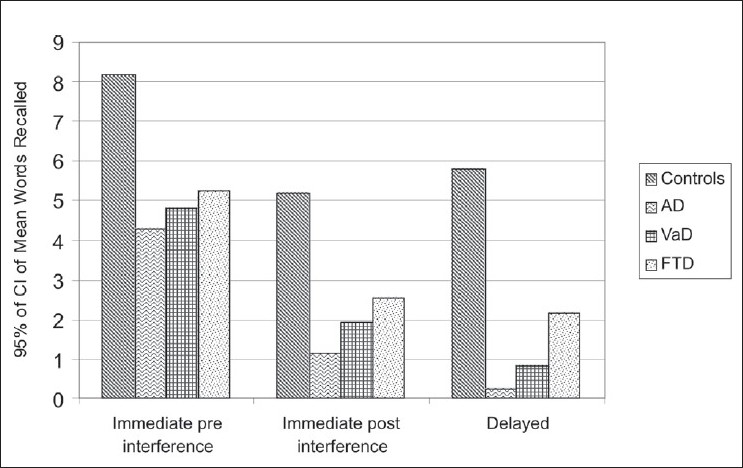
Comparison of recall: immediate pre-interference, immediate post-interference, and delayed

Figure 1 shows a decline in the mean words recalled immediately after interference in the controls and the patient groups. This decline in mean words recalled was significant in all the three dementia groups as well as in the controls (*P* < 0.01)

On the delayed recall, when compared to the immediate post-interference, recall of words was impaired in the patient group (*P* < 0.05) but not in the controls.

**Figure 2:**
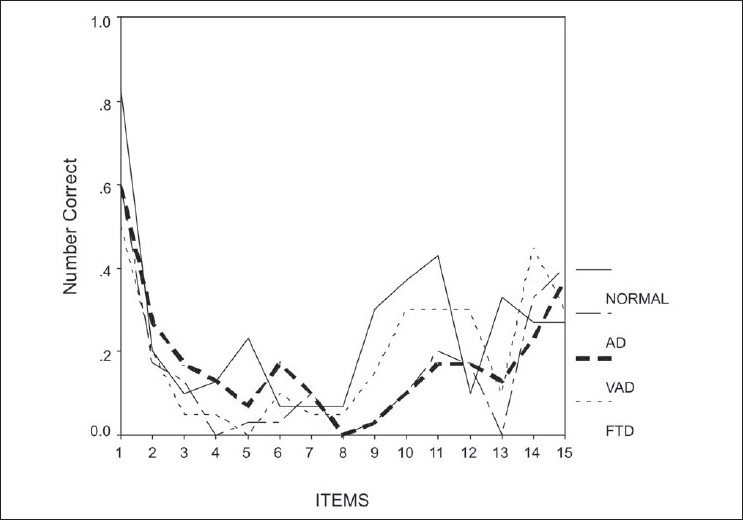
Graph showing the serial position effect in the free recall of 15 items in the list

Figure 2 demonstrates the phenomenon of SPE in free recall for all the 15 items in the list. The pattern obtained for the patient group was similar to that for the control group.

**Figure 3:**
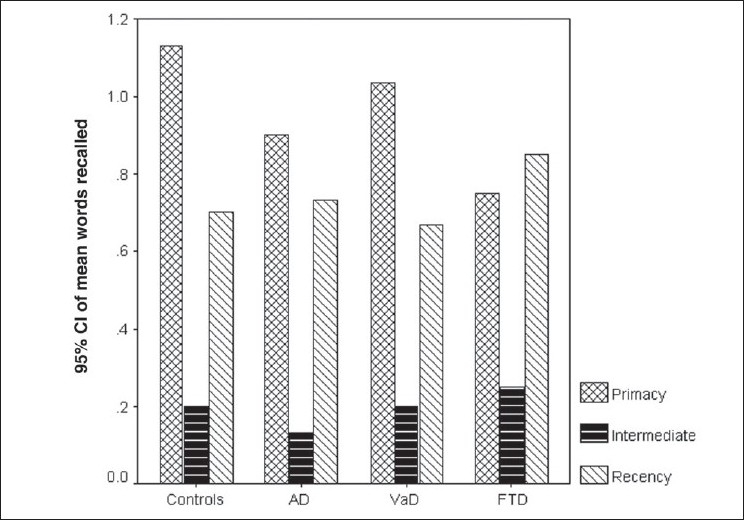
Graph showing the primacy–recency effect for the free recall for each of the groups

Figure 3 demonstrates the primacy/recency effect in all the four groups. From the graph it can be seen that primacy and recency were preserved in the AD and VaD groups, just as in the control group. However, the FTD group demonstrated only the recency effect. Statistical analysis showed that the AD and VAD groups demonstrated significant primacy and recency effects, similar to that seen in the controls (*P* < 0.01 using Bonferroni correction). The FTD group showed a significant recency effect compared to intermediate recall (*P* < 0.01 using Bonferroni correction).

**Figure 4:**
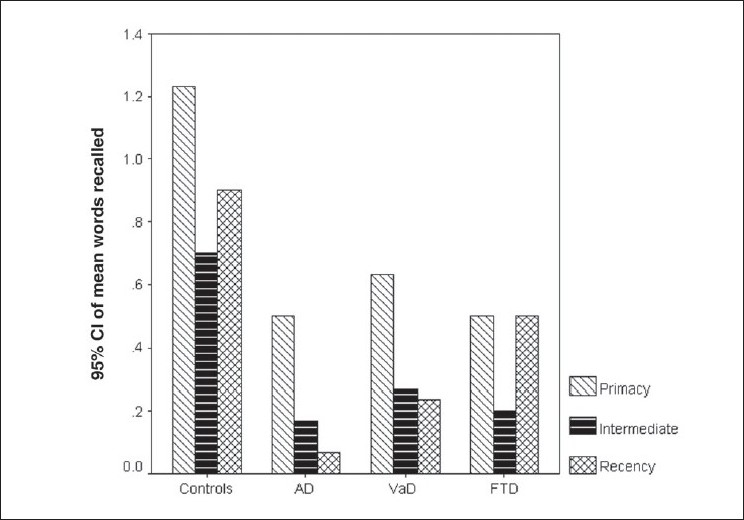
Graph showing the primacy–recency effect for the post-interference free recall for each of the groups

We also studied the pattern of SPE following interference to determine whether the pattern of words recalled following interference would be similar to that in free recall. Following interference, the pattern of the SPE was lost in dementia patients as well as in controls [Figure 4]. Within-group comparison showed a superior primacy effect in the control group (*P* < 0.05) compared to the intermediate. The AD group demonstrated a significantly better primacy effect compared to the intermediate (*P* < 0.05 using Bonferroni correction) and the recency effect (*P* < 0.01 using Bonferroni correction). The VaD group demonstrated a superior primacy effect compared to the recency effect. The FTD group demonstrated the pattern of SPE; seen as only a trend. Between-group comparisons showed that when compared to controls, the AD and VaD groups demonstrated impaired primacy, intermediate, and recency effects (*P* < 0.05 using Bonferroni correction). As compared to the control group, the FTD group demonstrated impaired primacy and intermediate effect (*P* < 0.05 using Bonferroni correction) but was comparable with the control on the recency effect. On SPE there was no difference seen among the dementia groups.

**Figure 5:**
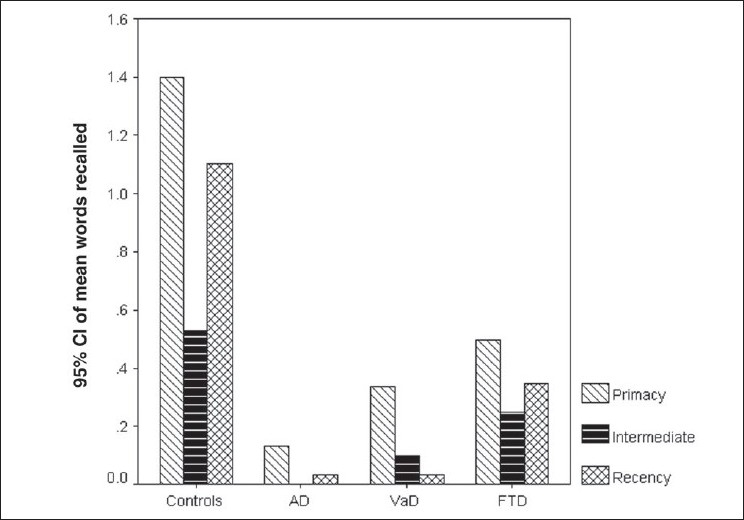
Graph showing the primacy–recency effect for the delayed recall for each of the groups

Figure 5 shows the pattern of word recall on the delayed recall trial. During delayed recall, although the pattern of SPE was preserved in the control and FTD groups, only the controls demonstrated significant primacy and recency effects (*P* < 0.01 using Bonferroni correction). While the significant primacy effect seen in the FTD group was in comparison to the intermediate (*P* < 0.05 using Bonferroni correction), in the VaD group the superior primacy was in comparison to the recency effect (*P* < 0.05 using Bonferroni correction). Recall of words was not seen to follow the phenomenon of SPE in the AD group.

## Discussion

Studies on the pattern of learning and recall in patients with dementia are scarce. The few available studies have focused only on AD and VaD. To the best of our knowledge, there are no published studies on the pattern of learning and recall in patients with FTD, or studies comparing the three types of dementias with controls.

In our study we found that dementia patients recalled fewer words than normal controls. Recall of fewer words in dementia patients is possibly attributable to poor encoding of the stimuli presented to them.[17] Our study replicates the findings of Bayley *et al*.[18] who used the California Verbal Learning Test (CVLT) and found that AD patients recalled significantly fewer words than normal controls and had significantly reduced primacy effect, with a relatively preserved recency effect.

In the dementia groups, we have seen that although the recall of words is less than in the controls, there is an improvement in scores with successive trials. Our findings are consistent with the reports by Bigler *et al*.[19] The authors, based on their study of 94 AD patients on RAVLT, reported that AD patients had negligible improvement in the learning/retention curve with repeated trials. Burkart *et al*.[4] have also reported similar findings based on their study of 44 AD patients and 24 non-demented controls. Becker *et al*.[17] studied 62 patients with mild AD and 64 elderly controls and found that AD patients did not have an abnormal rate of forgetting; they concluded that poor initial encoding of the stimuli may be the cause of impaired recall in AD patients.

In the free recall and recognition task, the AD and VaD groups in our study were indistinguishable in the scores obtained. The results are similar to that reported by Almkvist *et al*.[20] In addition, in our study, the mean recall by the FTD group in the pre-interference trial was similar to the recall by the AD and VaD groups.

On the post-interference and delayed recall trials, the three dementia groups remained comparable on the mean number of words recalled, suggesting that interference profoundly hampers the process of retrieval, independent of the type of dementia.

Nevertheless, in the patient groups there was a significant improvement in scores on the recognition task when compared to that on the recall task, which suggests that some information is indeed still accessible for recognition though not for free recall. On the recognition task, the FTD group had more false positives than all other groups (*P* < 0.01). The reason is possibly attributable to a greater tendency to perseverate with the 'yes' response. In contrast, the AD and VaD groups demonstrated more misses compared to the other two groups (*P* < 0.01). The fact that these groups did not have more false positives indicates that their efforts are not just random guesses but that recognition is indeed assisting the retrieval of information that is inaccessible for free recall. These results suggest that in the dementia groups encoded information is not completely lost (or disintegrated). Instead, either its tagging is lost or disrupted or, alternatively, the neural network(s) responsible for free recall is/are structurally disintegrated.

In this study, we have also focused on the qualitative aspect of the SPE phenomenon in demented and cognitively unimpaired individuals. In the cognitively unimpaired, the normal pattern of SPE of recency being superior to primacy is well established in the works of early researchers like Nipher,[3] Deese and Kaufman,[21] Jahnke,[22] and Rundus.[23] The reason for a superior recency effect in these individuals has been reported by researchers like Raaijmakers and Shiffrin,[24,25] and Gillund and Shiffrin.[26] In cognitively unimpaired individuals, items toward the end of the list are recalled better in free recall because the contents of short-term storage are available for free recall at the time of the test and also, perhaps, due to the retroactive interference during encoding, i.e., the earlier items suffer interference from the later ones in the list.[27]

Our results in patients with dementia show that, as in controls, the SPE is preserved in free recall in the AD and VaD groups. However, none of the dementia groups showed a superior recency effect compared to the primacy effect. These findings are consistent with that of Pepin and Eslinger[5] who also showed that in mild AD both primacy and recency were above the intermediate portion of the curve and the SPE is 'U' shaped as it is in normal individuals. They also report a fading of the primacy effect with increasing severity of dementia. However, the results in the AD group are in contrast to that found in other studies, which have indicated impaired primacy in AD patients.[6] In our study, the age of our patients could have contributed to the better primacy effect. The AD patients in our study are distinctly younger (68.93 ± 7.28 years) than the patients in the study by Buschke *et al*. (80.6 ± 6.4 years). Thus it is possible that with regard to SPE there may be an interaction between age and dementia.

Our results in the VaD group showed an SPE with superior primacy and recency effects compared to intermediate (*P* < 0.01). A similar study by Paul *et al*.[8] demonstrated an intact SPE for patients with mild VaD.

In our study, the FTD group demonstrated a superior recency effect compared to intermediate (*P* < 0.01), which could be accounted for by the fact that in FTD the central executive system in the frontal lobes may be functioning at slightly impaired levels. The three-component model of working memory by Baddeley and Hitch[28] explains the finding seen in FTD. As per the phonological loop storage system in their model, as the number of items in a list that need to be rehearsed increases, what happens is that before the first item can be rehearsed it fades out of the memory storage.[29] According to Hashimoto *et al*.,[30] to learn the words efficiently, participants should inhibit the words already learnt, selectively attend to the unrecalled words, and actively rehearse them. The manipulation of the information and attention shifting is known to be the function of the central executive system. As this function may be impaired in FTD, it is possible that FTD patients are unable to inhibit the retroactive interference during serial learning trials.

Thus, the results of our study suggest that while, quantitatively, memory storage and retrieval is ravaged by dementing diseases, the organization of memory-encoding mechanisms in patients with dementia seems to be less afflicted by the disease in the early stages and remains largely the same as in cognitively unimpaired individuals. Qualitatively, the mechanisms of encoding are relatively preserved in mild dementia, though the mechanisms of free retrieval and cued retrieval are differentially impaired. In addition, between the controls and dementia groups, there are differences seen in the quantitative aspects of encoding and/or retrieval.

Based on the findings of this study, we conclude that quantitative learning is affected by dementia. However, the pattern of qualitative learning, as measured by the SPE in free recall, remains largely unaltered by dementia in the early stages, suggesting that this type of learning is not affected by mild dementia.
